# Postconditioning with Lactate-Enriched Blood for Reducing Lethal Reperfusion Injury in Humans

**DOI:** 10.1007/s12265-023-10372-y

**Published:** 2023-03-20

**Authors:** Takashi Koyama

**Affiliations:** Department of Cardiology, Saitama Municipal Hospital, 2460 Mimuro, Midori-Ku, Saitama City, Saitama 336-8522 Japan

**Keywords:** Cardioprotection, Ischemia reperfusion injury, ST-segment elevation myocardial infarction

## Abstract

Ischemic myocardium cannot survive without reperfusion. However, reperfusion of the ischemic myocardium paradoxically induces myocyte death; this phenomenon is termed lethal reperfusion injury. To date, no effective approach has been demonstrated for ST-segment elevation myocardial infarction (STEMI) in clinical settings. Recently, we demonstrated a novel approach for cardioprotection, termed postconditioning with lactate-enriched blood (PCLeB). PCLeB comprises intermittent reperfusion and timely coronary injections of lactated Ringer’s solution, which is implemented at the beginning of reperfusion. This approach is aimed at reducing lethal reperfusion injury via prolonging intracellular acidosis during the early period of reperfusion, compared with the original postconditioning protocol. Patients with STEMI treated using PCLeB have reported positive outcomes. This article represents an effort, with a perspective different from current insights, toward preventing lethal reperfusion injury, in light of the historical background of reperfusion injury research. PCLeB is considered the new approach for cardioprotection.

**Graphical Abstract:**

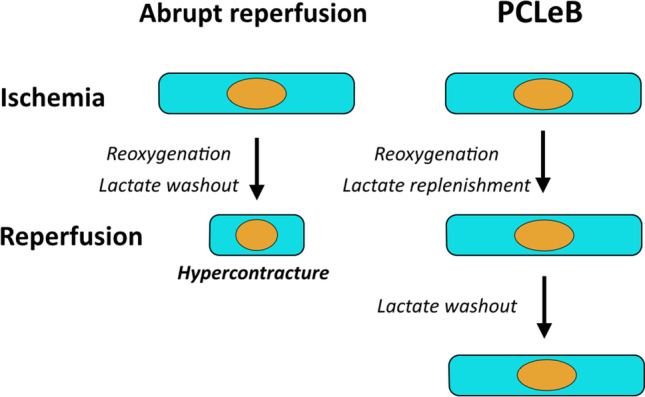

## Introduction

Ischemic myocardium cannot survive without reperfusion; thus, the timely restoration of coronary blood flow to the occluded coronary artery is vital for salvaging the myocardial cells from ischemic cell death. However, myocardial salvage by coronary reperfusion is achieved at the expense of various deleterious effects termed as myocardial reperfusion injury, which attenuates the beneficial effects of reperfusion therapy for ST-segment elevation myocardial infarction (STEMI). This is an established but still unresolved issue that needs to be overcome in order to achieve better outcomes in patients with STEMI.

In previous myocardial reperfusion injury research, Jennings et al. first described that reperfusion is not only beneficial but also harmful to the ischemic myocardium [1, 2], using a canine model of ischemia and reperfusion in the 1960s. They reported the characteristic features observed in a reperfused ischemic myocardium, such as widespread contraction-band with sarcolemmal disruptions, translocation and disorganization of the mitochondria, and large dense bodies (calcium deposits) in the mitochondria, which were different from and considerably more extensive than those produced by equivalent periods of permanent ischemia. The 1980s were the era of reperfusion therapy for ST-segment elevation myocardial infarction (STEMI). Concurrently, myocardial reperfusion injury emerged as a challenge to clinicians who treated patients with STEMI. In the early 1990s, based on the insights from experimental studies and clinical experience, myocardial reperfusion injury was delineated and classified into four types—myocardial stunning, reperfusion arrhythmia, microvascular reperfusion injury, and lethal reperfusion injury [3]. This standard classification is valid in the current era. Both myocardial stunning and reperfusion arrhythmia are self-limiting and, therefore, can be managed without difficulties. In contrast, microvascular reperfusion injury, which is clinically observed as slow-flow or no-reflow following reperfusion therapy, and lethal reperfusion injury, which reduces the myocardial salvaging effects of reperfusion therapy by inducing myocardial cell death in reversibly injured cells, adversely affect the outcomes in patients with STEMI. Therefore, these two injuries have greater clinical importance. A lethal reperfusion injury enlarges the myocardial infarct size and directly affects the outcomes in patients with STEMI; therefore, its prevention is particularly important. Various approaches have been verified in the clinical settings to prevent lethal reperfusion injury, based on the successful results of experimental studies. However, no approach has been successful in preventing this injury to date [4]. Therefore, research for preventing lethal reperfusion injury appears to be at a deadlock. This article represents an effort, with a perspective different from the current insights, to explore an effective approach for preventing lethal reperfusion injury, along with a review of the historical research in lethal reperfusion injuries.

## Lethal Reperfusion Injury in Humans

There has been an ongoing debate regarding lethal reperfusion injury in humans. Lethal reperfusion injury refers to the forced transition of reversibly injured cells into irreversibly injured cells following reperfusion. To date, researchers have not identified histological changes indicating the beginning of irreversible alterations in the ischemic myocardium. This is principally because histological observations of serial specimens obtained from a similar individual cannot be performed during ischemia–reperfusion sequence even in animal experiments. Therefore, it is unclear if a portion of the infarcted myocardium remained reversibly injured at the end of ischemia. The only way to corroborate the presence of lethal reperfusion injury is to demonstrate a reduction in the infarct size achieved by interventions implemented not before ischemia but only upon/following reperfusion. Various animal studies have demonstrated a reduction in the infarct size by pharmacological agents [5–9] or controlled reperfusion, such as ischemic postconditioning [10]. Despite some success in small pilot studies, no approach has been successful in demonstrating a reduction in the infarct size or improved outcomes in large-scale trials in humans [4]. Therefore, lethal reperfusion injury has still not been confirmed in humans. Nevertheless, because of its massive clinical importance, several investigators and clinicians are enthusiastically pursuing an effective approach to prevent lethal reperfusion injury for reducing the infarct size and improving the outcomes in patients with STEMI.

## Historical Transition of the Leading Hypothetical Mechanism Underlying Lethal Reperfusion Injury

### Ca^2+^ Influx via Na^+^/Ca^2+^ Exchangers

In 1972, Shen and Jennings demonstrated a marked increase in the tissue Ca^2+^ uptake and calcium content in the reperfused ischemic myocardium in dogs; furthermore, the majority of calcium was localized in dense bodies in the mitochondria [11, 12]. They suggested that this large Ca^2+^ influx may play an important role in irreversible cell injury. Subsequently, they made notable efforts to elucidate the mechanism underlying this uncontrolled influx of Ca^2+^ with regard to lethal reperfusion injury. The dominant hypothetical theory during the 1980s through 1990s was as follows. Upon reperfusion, excessive intracellular H^+^ accumulated during ischemia is extruded via Na^+^/H^+^ exchangers in exchange for Na^+^. Consequently, this Na^+^ is extruded via Na^+^/Ca^2+^ changers in exchange for Ca^2+^, and the resultant large Ca^2+^ influx leads to an irreversible cell injury [13, 14]. In the 2000s, researchers accomplished large-scale clinical trials in which they administered specific inhibitors of Na^+^/H^+^ exchangers, such as eniporide [15] or caliporide [16], in patients with STEMI before reperfusion to examine their cardioprotective effects. The results demonstrated no benefit in terms of the infarct size or clinical outcomes, which highlighted the need to explore an alternative mechanism primarily responsible for lethal reperfusion injury.

### Mitochondrial Permeability Transition (MPT)

In the 1990s, investigators demonstrated the possible involvement of mitochondrial permeability transition (MPT) in the mechanism underlying lethal reperfusion injury [17, 18]. The MPT pore is a nonselective channel on the inner mitochondrial membrane. It remains closed during ischemia and opens within the first few minutes of reperfusion, following induction by mitochondrial Ca^2+^ overload, oxidative stress, restoration of physiological pH, and adenosine triphosphate (ATP) depletion [18, 19]. Its opening leads to the collapse of the mitochondrial membrane potential, the uncoupling of oxidative phosphorylation, and eventual cell death. This novel theory was gradually accepted. Following the failure of the clinical trials that used specific inhibitors of Na^+^/H^+^ exchangers in patients with STEMI, this theory became the leading hypothetical mechanism underlying lethal reperfusion injury.

## Recent Attempts to Prevent Myocardial Reperfusion Injury in Humans

### Ischemic Postconditioning

Ischemic postconditioning, which was originally reported by Zhao et al. following their successful experimental study in 2003 [10], involves brief episodes of intermittent reperfusion before complete reperfusion. Three brief episodes of intermittent reperfusion implemented immediately following 45 min of ischemia could significantly reduce the myocardial infarct size in dogs. This approach was rapidly applied to the clinical settings by Staat et al. in 2005 [20]. In their small pilot study involving 30 patients with STEMI, four brief cycles of intermittent reperfusion before complete reperfusion reduced the myocardial infarct size by 36%. This novel study demonstrated the presence of lethal reperfusion injury in humans because the intervention was performed only at the beginning of reperfusion. The cardioprotective effects of ischemic postconditioning have been attributed to the delayed recovery from intracellular acidosis during ischemia and, more precisely, to the attenuation of MPT pore opening owing to the delayed normalization of intracellular pH [21, 22]. However, a similar approach failed to improve the outcomes in patients with STEMI in large-scale clinical trials in the subsequent years [23–25].

### Cyclosporine-A

Animal models of ischemia and reperfusion demonstrated that cyclosporine-A—an immune-suppressant that inhibits MPT—reduces the myocardial infarct size up to 50% [9, 26, 27]. In 2008, Piot et al. translated this observation to the clinical settings in their small pilot study [28]. The administration of cyclosporine-A before primary percutaneous coronary intervention (PCI) reduced the myocardial infarct size by 40% in patients with STEMI. However, two recent large-scale clinical trials reported no improvements in the long-term outcomes—mortality and the incidence of heart failure—in patients with STEMI who were treated with prior cyclosporine-A administration and subsequent PCI [29, 30].

## Disrupting the Deadlock

Attempts to prevent myocardial reperfusion injury in humans have supposedly ended in a deadlock. Some researchers argue that fundamental differences between animal experiments and human studies, such as younger and healthier animals in animal experiments and comorbidities or the longer duration of ischemia in human studies, may have contributed to the failures in human studies that assessed postconditioning and cyclosporine-A administration [4, 31]. However, upon eliminating these differences in future clinical studies, it is unclear if the results can be applied to STEMI treatment in the real world. Moreover, a generalized use of these approaches appears unlikely. Incidentally, if the investigators review the complete history of reperfusion injury research and identify flaws in excluding possibilities about the involvement of a certain mechanism in the incidence of lethal reperfusion injury, the phenomenon is worth further investigation without any preconceptions. This may be a conventional means to break the deadlock.

## The Oxygen Paradox (Reoxygenation Injury) and Reperfusion Injury

The oxygen paradox refers to reoxygenation injury of a previously oxygen-depleted myocardium [32, 33]. This phenomenon is associated with the release of creatine kinase (CK) and is histologically characterized by myofibrillar hypercontracture and sarcolemmal disruptions. Serial histological observations and analyses of CK release during reoxygenation suggest that hypercontracture precedes its release [34]. Therefore, some researchers believe that myofibrillar hypercontracture is responsible for sarcolemmal disruption and cell death, despite the absence of direct evidence [35, 36]. This theory is supported by the fact that CK release was not observed in hypoxia-reoxygenation injury in isolated myocytes, which have no intercellular junctions and, therefore, are likely to endure strong mechanical force generated by hypercontracture without sarcolemmal disruption [37].

In a reperfused ischemic myocardium, investigators have observed histological indicators of the oxygen paradox, such as myofibrillar hypercontracture and sarcolemmal disruptions [1, 2]. Thus, strong mechanical force generated by hypercontracture may play an important role also in ischemia–reperfusion injury. A well-developed hypercontracture—histologically confirmed as contraction band necrosis—has been reported within 120 s of ischemic myocardium reperfusion [38]. Considering the MPT pore requires minutes to open following reperfusion, reperfusion-induced hypercontracture appears to precede MPT-related irreversible cell injury in the reperfused ischemic myocardium. This may be one possible explanation for the failure of cyclosporine-A to improve the outcomes in patients with STEMI. If reoxygenation/reperfusion-induced hypercontracture in the reperfused ischemic myocardium is the true cause of lethal reperfusion injury, clinicians should target hypercontracture for preventing the injury instead of targeting a sequence of events in the irreversibly injured myocardium. Such attempts have been made in an animal model of ischemia and reperfusion using 2,3-butanedione monoxime, a potent inhibitor of the myofibrils. The inhibition of the myofibrillar contractile force during reperfusion using 2,3-butanedione monoxime significantly reduces the infarct size in in-situ hearts of both pigs (by 31%) [5] and dogs (by 73%) [6]. Unfortunately, its application in humans is impossible because of its toxicity. However, these strongly positive results in experimental studies should not be overlooked.

## A Large Ca^2+^ Influx and Reperfusion-Induced Hypercontracture

Historically, reperfusion injury research gathered momentum when Shen and Jennings reported on a marked increase in the tissue Ca^2+^ uptake and calcium content in reperfused canine ischemic myocardium in 1972 [11, 12], thereby suggesting that a large Ca^2+^ influx during reperfusion may play an important role in irreversible cell injury. However, no evidence has clarified if this large Ca^2+^ influx is the primary cause of lethal reperfusion injury or is secondary to a preexisting irreversible injury to the cell structure. Ca^2+^ influx via the Na^+^/Ca^2+^ exchangers to extrude the excessive Na^+^ that was intruded into the cells via Na^+^/H^+^ exchangers against the excessive H^+^ accumulated during ischemia was the dominant hypothetical mechanism of this large Ca^2+^ influx during the 1980s through 1990s [13, 14]. This theory adheres to the idea that a large Ca^2^^＋^ influx during reperfusion is the primary cause of lethal reperfusion injury. However, it has lost reliability following the failure of the outcome trials that used specific inhibitors of Na^+^/H^+^ exchangers in patients with STEMI [15, 16]. Similarly, Ca^2+^ channel blockers administered during reperfusion in pharmacological concentrations failed to prevent Ca^2+^ gain during reperfusion in experimental studies [39, 40]; therefore, the Ca^2+^ channel is not the possible route of the large Ca^2+^ influx. Hence, the nonspecific entry of Ca^2+^ from the disrupted or permeable sarcolemma appears to be the only possible route for the large Ca^2+^ influx. Sarcolemmal disruption is one of the histological indicators of the oxygen paradox. Further, it is observed in reperfused ischemic myocardium, presumably caused by the strong mechanical force generated by hypercontracture. Therefore, the large Ca^2+^ influx may be a part of a sequence of deleterious events following irreversible cell injury. Considering the potent cardioprotective effects of 2,3-butanedione monoxime, reperfusion-induced hypercontracture is possibly responsible for lethal reperfusion injury.

## Acidic Reperfusion and Terminal Warm Blood Cardioplegia

Acidic reperfusion or perfusate is protective against ischemia–reperfusion or hypoxia-reoxygenation injury in in-situ hearts [7, 41] and isolated myocytes [37]. The prevention of Na^+^/H^+^ exchange or, more recently, the prevention of MPT was the supposed mechanism underlying the cardioprotective effects. However, such mechanisms may be questionable following the failure of the inhibitors of both mechanisms to improve the outcomes in patients with STEMI [15, 16, 29, 30]. An alternative mechanism is reduction in the ability of myofilaments to develop force via the inhibition of Ca^2+^ binding to troponin C through the competitive binding of H^+^ to troponin C [42]. This mechanism is relatively similar to that wherein 2,3-butanedione monoxime administration during early reperfusion reduced the infarct size by preventing myofibrillar force development [5, 6].

Another familiar cardioprotective approach implemented at the beginning of reperfusion is terminal warm blood cardioplegia, which is used in cardiac surgery worldwide. In the late 1980s, Buckberg and his coworkers demonstrated that reperfusion for 20 min to 30 min with hyperkalemic blood prevented contraction and reduced myocyte deaths during totally vented bypass in canine hearts [43, 44]. This finding has been translated to open-heart surgery in humans. During open-heart surgery, the heart is halted using potassium arrest. In terminal warm blood cardioplegia, this arrest is extended into the early reperfusion period using reperfusion with warm blood comprising high-potassium concentrations. This approach prevents the restoration of heartbeat during the early reperfusion period. Moreover, this approach is relatively similar to cardioprotection using 2,3-butanedione monoxime in terms of preventing myofibrillar force development during the early reperfusion period.

Therefore, the blockade of contractile activity during the early reperfusion period—which may prevent hypercontracture development in the reperfused ischemic myocardium—appears to protect the myocardium against reperfusion injury. The approach that can prevent myofibrillar force development during the early reperfusion period in STEMI treatment is questionable. Both acidic reperfusion—adding hydrochloric acid to the reperfusing blood—and 2,3-butanedione monoxime cannot be used in humans. Furthermore, potassium arrest cannot be used in STEMI treatment.

## Postconditioning with Lactate-Enriched Blood (PCLeB)

**Fig. 1:**
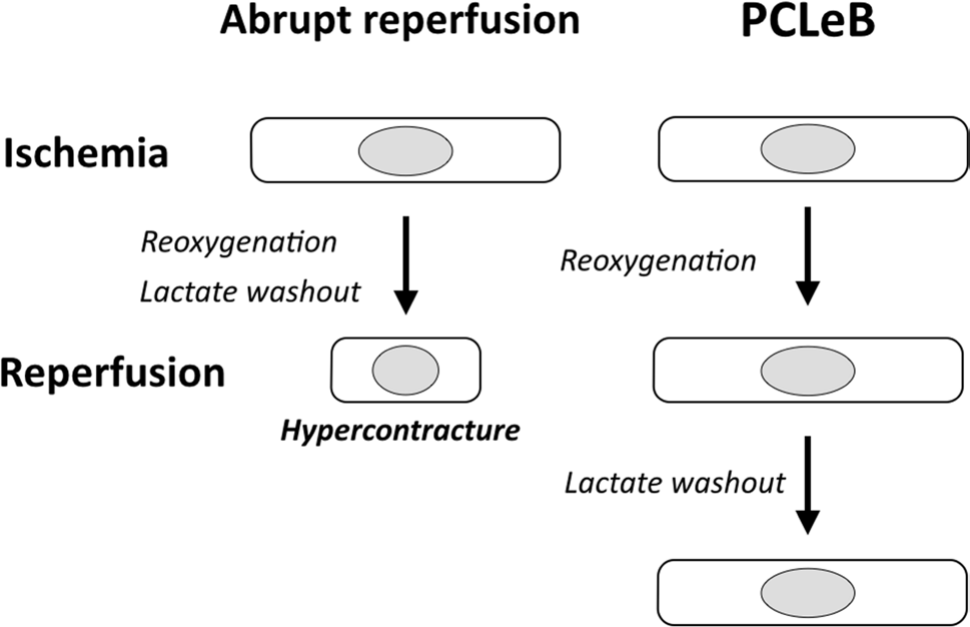
A schema of the hypothesis for the prevention of reperfusion-induced hyperontracture using PCLeB. Left panel: Abrupt reperfusion causes the hypercontracture of myocardial cells. Right panel: In case of reperfusion using PCLeB, hypercontracture can be prevented by suspending the lactate washout during the early reperfusion period. PCLeB, postconditioning with lactate-enriched blood

The ischemic myocardium ceases contraction to retain ATP by accumulating lactate, thus producing intracellular acidosis; therefore, lactate works as an inherent contractile activity blocker during ischemia. The presence of lactate inside the myocardium during the early reperfusion period attenuates myofibrillar force recovery, and expectedly prevents hypercontracture (Fig. 1). This phenomenon is similar to prolonging the potassium-arrest period, as overlapping the early reperfusion period prevents the restoration of heartbeat at reperfusion and protects the reperfused myocardium in terminal warm blood cardioplegia. An ischemic postconditioning delays lactate washout and prolongs intracellular acidosis. However, it fails to improve the outcomes in patients with STEMI, suggesting that the delay in lactate washout is insufficient, despite the approach being strategically accurate.

**Fig. 2:**
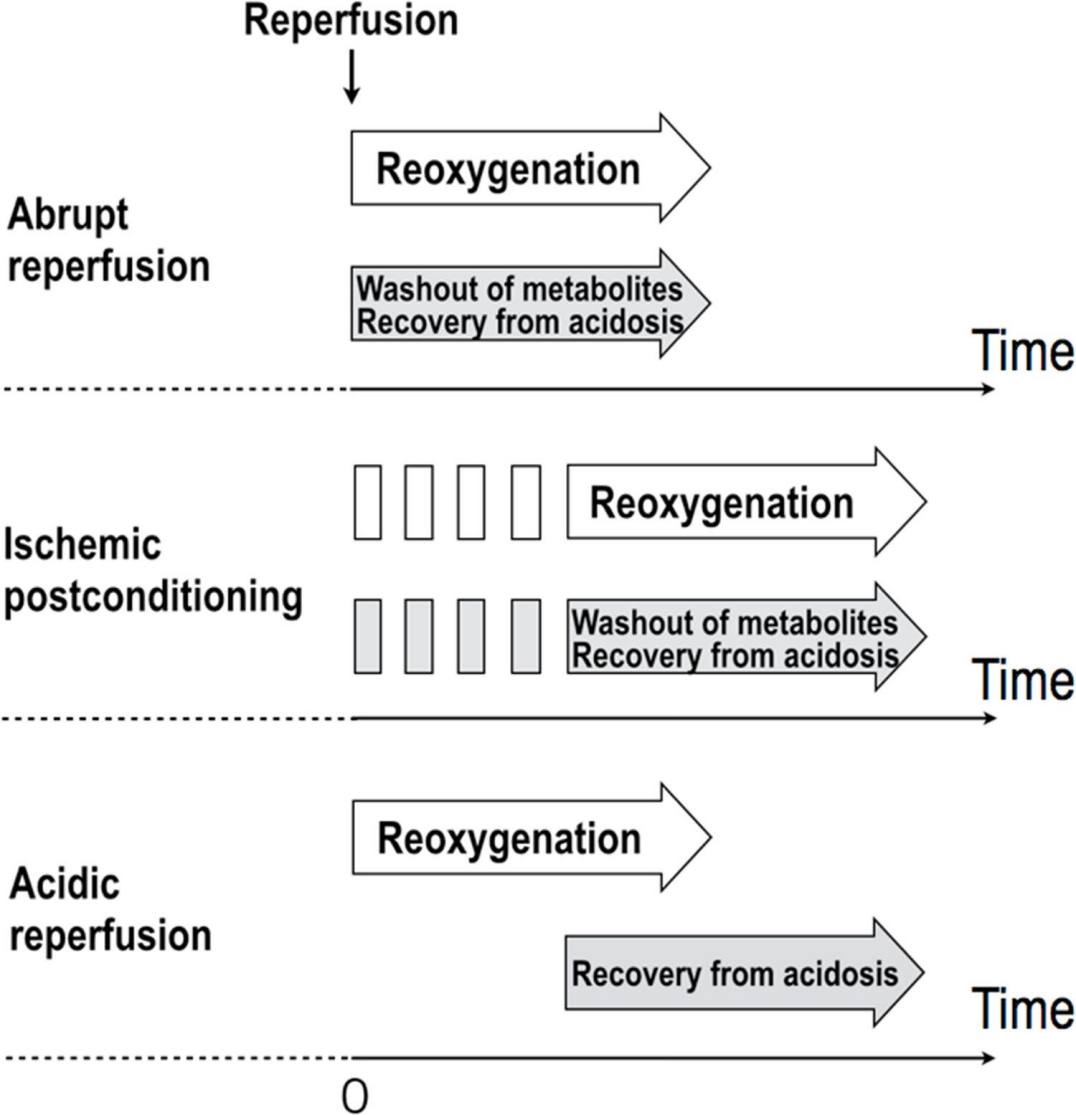
Schematic representation of the difference between ischemic postconditioning and acidic reperfusion in terms of the mode of prolonging intracellular acidosis. Abrupt reperfusion simultaneously induces the reoxygenation of the tissue and the washout of metabolites, including lactate, which is primarily responsible for intracellular acidosis during ischemia (upper panel). Ischemic postconditioning delays both reoxygenation and recovery from intracellular acidosis on a similar time scale (middle panel). In contrast, acidic reperfusion selectively delays the recovery from intracellular acidosis (bottom panel). Reprinted from Koyama and Akima [58] with black and white conversion

**Fig. 3:**
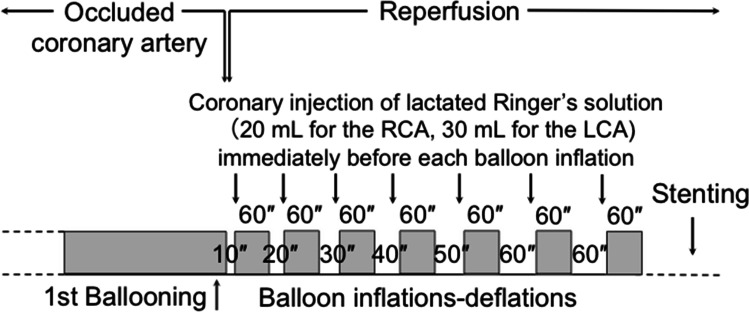
An overview of the protocol for postconditioning with lactate-enriched blood. The duration of each brief reperfusion is prolonged from 10 to 60 s in succession. At the end of each brief reperfusion, lactate is supplied by injecting lactated Ringer’s solution into the target coronary artery. The balloon is rapidly inflated at the lesion site to trap the lactate inside the ischemic myocardium. Each brief ischemic period lasts for 60 s. Following seven cycles of balloon inflation and deflation, full reperfusion is performed, followed by stenting. LCA, left coronary artery; RCA, right coronary artery. Reprinted from Koyama et al. [46]

In the view of prolonging intracellular acidosis, intermittent reperfusion delays the recovery from intracellular acidosis. However, it is unclear if intermittent reperfusion delays the recovery physiologically. Reoxygenation, replenishment of substrates, and washout of metabolites (e.g., lactate) occur simultaneously upon reperfusion. Intermittent reperfusion decelerates these processes that reestablish the physiological state on a similar time scale. In contrast, acidic reperfusion selectively delays the recovery from intracellular acidosis (Fig. 2). Postconditioning, the failure of “intermittent reperfusion” to improve the outcomes in patients with STEMI can be explained by the inability of this maneuver to selectively delay recovery from intracellular acidosis. To produce identical cardioprotective effects as that of acidic reperfusion in STEMI treatment, lactate washout—whose accumulation is principally responsible for intracellular acidosis during ischemia—requires further delay relative to the rate of re-oxygenation during postconditioning. PCLeB was based on this concept [45–48] (Figs. 1 and 3). To further delay the lactate washout, timely coronary injections of lactated Ringer’s solution were incorporated into “intermittent reperfusion” postconditioning in PCLeB. Keeping extracellular lactate concentrations high through PCLeB should attenuate the extrusion of intracellular lactate via the monocarboxylate transporter, thus prolonging intracellular acidosis and hampering force development of the myofilaments. In the PCLeB protocol, the duration of each brief reperfusion is prolonged in succession from 10 to 60 s to prevent the rapid and abrupt washout of lactate in the early phase of reperfusion. At the end of each brief reperfusion, the lactate washed away during each brief reperfusion is replenished in the reperfused myocardium by a coronary injection of lactated Ringer’s solution. Further, the balloon is rapidly inflated at the lesion site, such that the lactate is trapped within the ischemic myocardium during each brief, repetitive episode of ischemia. This approach facilitates attaining a higher level of tissue lactate concentration for the entire duration of postconditioning; therefore, a significant attenuation of contractile force recovery can be expected due to the prolonged intracellular acidosis during the procedures.

PCLeB is apparently effective against all four types of myocardial reperfusion injuries—reperfusion arrhythmia [49], myocardial stunning [50, 51], microvascular reperfusion injury [46, 52], and lethal reperfusion injury [47]. Furthermore, despite the small-scale nature of the studies, they reported on good short- [50] and long-term [53] outcomes associated with reduced plasma NT-probrain natriuretic peptide (BNP) levels in patients with STEMI treated using PCLeB. Furthermore, PCLeB had been associated with a lesser increase in C-reactive protein (CRP) in patients with STEMI, compared to the matched control, in our matched case–control study [46], which may contribute to good outcomes in patients with STEMI since excessive early inflammation after myocardial infarction has been associated with adverse left ventricular remodeling [54, 55] and poor outcomes [56].

**Fig. 4:**
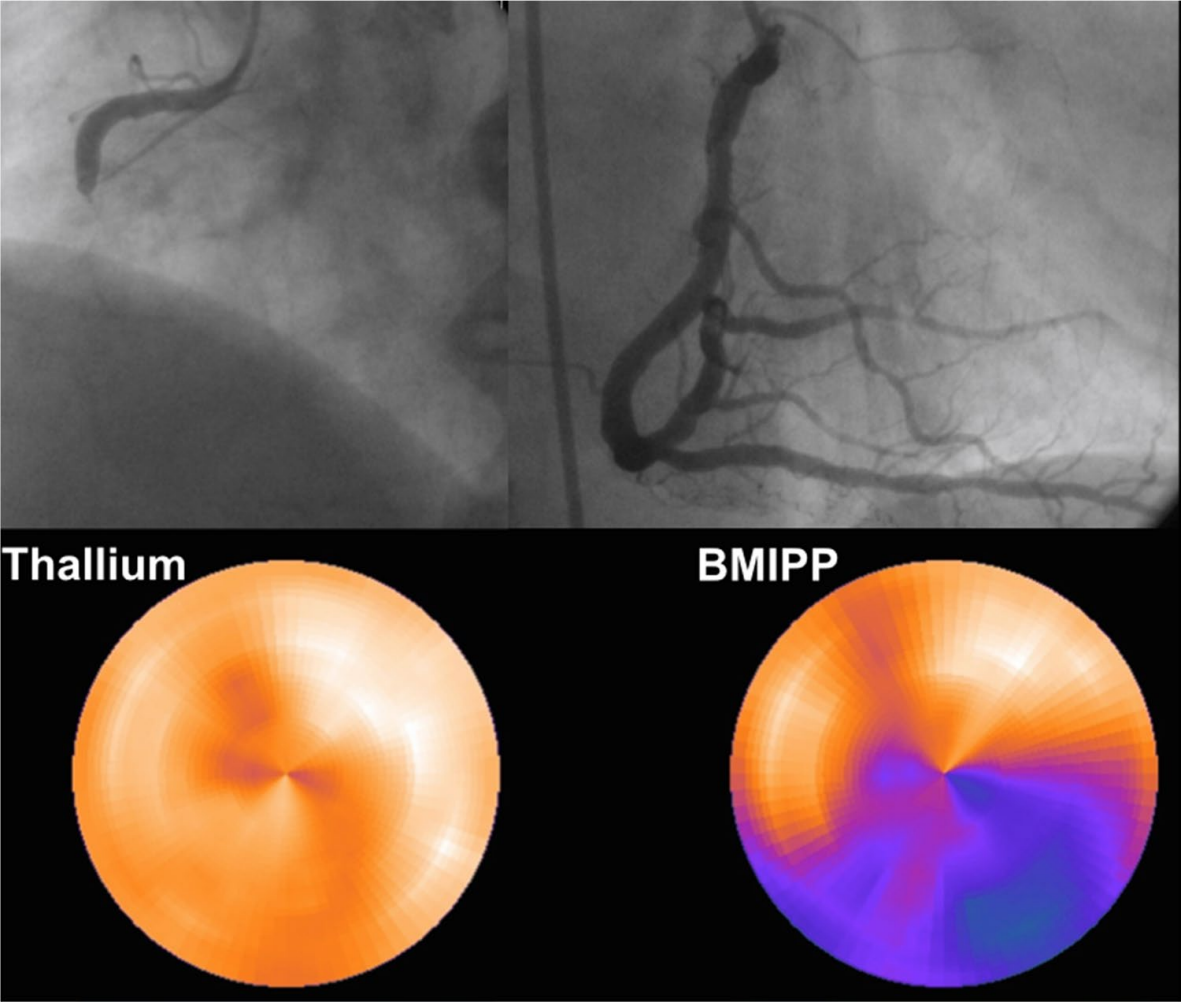
Coronary angiography (CAG) before and after the completion of primary coronary intervention (PCI) and dual-loading scintigraphy with thallium-201 and iodine-123 BMIPP before discharge. Upper left panel: CAG prior to PCI. The proximal right coronary artery (RCA) is completely occluded. Upper right panel: CAG after completion of PCI. Lower panel: Bull’s eye displays of dual-loading scintigraphy with thallium-201 and iodine-123 BMIPP before discharge. The take-up of thallium by the myocardium supplied by the RCA was excellent, in stark contrast to the large defect observed in BMIPP imaging. Reprinted from Koyama et al. [57]

Figure 4 shows a coronary angiogram before and after the completion of PCI using PCLeB and dual-loading scintigraphy with thalium-201 and iodine-123 BMIPP before discharge, as previously reported in a representative case presentation [57]. A 65-year-old male patient, presenting with 5 h of prolonged chest pain, underwent coronary angiography, which revealed total occlusion of the proximal right coronary artery with second-degree collateral circulation supplied by the left coronary artery. PCI using PCLeB was successfully performed, and the peak CK and CK-MB values were 2003 IU/L and 180 IU/L, respectively. Resting dual-loading scintigraphy revealed an extensive discrepancy in tracer uptake within the right coronary artery area, suggesting the potent myocardial salvaging effects of PCLeB.

Recently, Koyama and Akima demonstrated the outcomes of 100 consecutive patients with STEMI treated using PCLeB (Table 1) [58]. In-hospital deaths were absent and associated with substantially low peak CRP levels. Furthermore, no patient died or was rehospitalized for heart failure during the 1-year follow-up, associated with substantially low NT-proBNP levels in the chronic phase.

**Table 1:** Clinicodemographic characteristics and outcomes of 100 consecutive patients with STEMI treated using PCLeB

Age (yr) (range)	64.7 ± 13.3 (30–92)
Male sex, *n*	77
Hypertension, *n*	58
Dyslipidemia, *n*	61
Diabetes mellitus, *n*	35
Time to reperfusion (h)	4.1 ± 2.5
Occluded coronary artery, *n*	
Left anterior descending artery	41
Left circumflex artery	18
Right coronary artery	41
Peak creatine kinase level (IU/L)	2873 ± 2036
Peak creatine kinase-MB level (IU/L)	267 ± 167
Peak C-reactive protein level (mg/dL) (*n* = 91)*	3.94 ± 3.71
Corrected TIMI frame count	22.8 ± 16.8
NT-proBNP level in the chronic phase (pg/mL) (*n* = 84)^†^	247 ± 312
In-hospital deaths, *n*	0
Diuretic use at discharge, *n*	1
Diuretic use at one year, *n*	3
Deaths within one year,* n*	0
Rehospitalization for heart failure within one year, *n*	0

*Nine patients with extracardiac inflammatory disorders were excluded from the analysis

^†^Fourteen patients aged ≥ 80 years were excluded from the analysis. Data were missing in two additional patients aged < 80 years

*PCLeB* postconditioning with lactate-enriched blood, *STEMI* ST-segment elevation myocardial infarction, *BNP* brain natriuretic peptide, *TIMI* thrombolysis in myocardial infarction

Reprinted from Koyama and Akima [58]

## Pharmacological Postconditioning

Despite various pharmacological agents being administered at the time of reperfusion having been shown to have cardioprotective effects in experimental animals [59], none of these agents, including cyclosporine-A [29, 30], have been successfully implemented in clinical situations in terms of improving outcomes or reducing infarct size. However, certain volatile anesthetics may be possible candidates for clinical implementation because of their established cardioprotective effects and extensive clinical usage in general anesthesia. For example, in animal models of ischemia and reperfusion, both sevoflurane and desflurane, when administered during reperfusion, reportedly reduced the infarct size and were associated with a lesser increase in the left ventricular end-diastolic pressure following reperfusion [60]. In this study, reperfusion-induced hypercontracture was attenuated by the negative inotropic properties of these volatile anesthetics. Thus, the cardioprotective effects of these anesthetics appear to rely on the same processes as the aforementioned cardioprotective approaches, such as the PCLeB. However, the myocardial and respiratory depressant profile of these anesthetic agents constitutes a major disadvantage in their potential clinical application as adjuvant treatment for patients with STEMI.

Instead of lactated Ringer’s solution, lactic acid has been successfully used in experimental studies. Specifically, it has been demonstrated that lactic acid and hydrogen-rich saline, administered at the time of reperfusion, reduce the size of the infarct in a rat model of ischemia–reperfusion injury [61]. The results of this study may provide basic evidence supporting the cardioprotective effects of PCLeB.

## Conclusions

Targeting reperfusion-induced hypercontracture appears to be the appropriate method to prevent lethal reperfusion injury. In applying this strategy to STEMI treatment, a contractile activity blocker must be safe and able to achieve a sufficiently high tissue concentration at the beginning of reperfusion. To meet these requirements, using lactate as an inherent contractile activity blocker is supposedly the only applicable approach in STEMI treatment. This is because lactate is safe and would have already accumulated in high concentrations in the ischemic myocardium before reperfusion. PCLeB was designed considering these advantages of lactate. In PCLeB, lactate—both accumulated and injected—is expected to prevent rapid contractile force recovery and, possibly, hypercontracture development in the reperfused ischemic myocardium.

Despite positive outcomes, previous studies investigating the cardioprotective effects of PCLeB were uncontrolled or nonrandomized controlled studies. Thus, multicenter randomized controlled trials are warranted to confirm the exact cardioprotective effects of PCLeB.

## Data Availability

Further information on the data and methodologies will be made available by the author as requested.

## Abbreviations

ATP: Adenosine triphosphate
BNP: Brain natriuretic peptide
CK: Creatine kinase
MPT: Mitochondrial permeability transition
PCI: Percutaneous coronary intervention
PCLeB: Postconditioning with lactate-enriched blood
STEMI: ST-segment elevation myocardial infarction
